# Palliative care education in undergraduate medical and nursing programs in Colombia: a cross-sectional analysis

**DOI:** 10.1186/s12904-024-01477-5

**Published:** 2024-06-13

**Authors:** Miguel Antonio Sánchez-Cárdenas, Camila Andrea Navarro Tibaquirá, Nidia Mantilla-Manosalva, David Andrade Fonseca, Alexandra Marin Morales, Martha Ximena León Delgado

**Affiliations:** 1 School of Nursing, Universidad El Bosque. Bogotá D.C. Colombia, Bogotá, D.C Colombia; 2 School of Medicine, Universidad de La Sabana, Bogotá, D.C Colombia

**Keywords:** Undergraduate medical education, Undergraduate nursing education, Palliative care, Palliative medicine

## Abstract

**Background:**

The number of people suffering from chronic diseases requiring palliative care (PC) is increasing rapidly. Therefore, PC teaching in undergraduate health science programs is necessary to improve primary PC based on international recommendations and available scientific evidence.

**Methods:**

A descriptive cross-sectional study was conducted. Active undergraduate medical and nursing programs that were approved by the Colombian Ministry of Education and integrated PC teaching into their curricula were included in the study. The total sample consisted of 48 programs: 31 nursing and 17 medical programs.

**Results:**

PC competencies are distributed throughout the curriculum in 41.67% of programs, in elective courses in 31.25%, and in mandatory courses in 27.08% of the programs. The average PC teaching hours is 81 for nursing and 57.6 for medicine. PC clinical rotations are not offered in 75% of the programs. For undergraduate nursing programs, the most frequent competencies taught are the definition and history of PC and identifying common symptoms associated with advanced disease. In undergraduate medicine, the most common competencies are pharmacological and non-pharmacological pain management and identification of PC needs.

**Conclusions:**

PC teaching in undergraduate health science programs mainly addresses the conceptual and theoretical aspects of PC, which are part of the competencies present throughout the programs’ curricula. Low availability of PC clinical rotations was identified. Future studies should assess whether the low availability of clinical rotations in PC limits the ability of students to develop the practical competencies necessary to provide quality PC.

**Trial registration:**

Not applicable.

**Supplementary Information:**

The online version contains supplementary material available at 10.1186/s12904-024-01477-5.

## Introduction

Currently, approximately 40 million people worldwide require palliative care (PC), 78% of whom live in low- or middle-income countries [1]. The World Health Organization (WHO) and the Latin American Palliative Care Association (ALCP) recommend the implementation of local policies and strategies to strengthen the capacity of human resources for health, as well as the inclusion of PC in the basic curricula of all healthcare programs and the education of volunteers and the general public [2]. According to the Colombian Palliative Care Observatory (OCCP) and the Colombian Ministry of Health, there are insufficient health professionals trained in PC in Colombia, and they urge to increase the number of undergraduate programs offering PC training [3–5] and to expand continuing education programs to strengthen the training of professionals in palliative medicine [6]. In recent years, several universities have included PC training in undergraduate medical, nursing, and psychology programs, and there are already strategies in place to adapt the content in the different programs to develop PC competencies [4, 7, 8]. At the first meeting of the Colombian Palliative Care Education Network (REDCOLEDUPAL) in 2015 [8], a list of competencies was proposed for undergraduate medical and nursing programs, framed within the recommendations of the International Association for Hospice & Palliative Care (IAHPC) in the List of Essential Practices in PC [9] and the recommendations [10] and core competencies in PC of the European Association for Palliative Care (EAPC) [11].

This background illustrates the transition from informal education to formal education programs that train professionals to manage symptoms associated with chronic, degenerative, and irreversible diseases, and enable them to develop skills required for interdisciplinary work [12]. There is little information about the PC competencies taught in the undergraduate programs offered in Colombia. Therefore, this study reports on the status of PC instruction in the country and contributes to future recommendations for developing PC competencies through PC teaching.

## Methods

### Study design

A descriptive cross-sectional study was conducted to characterize the PC teaching structure and PC competencies in undergraduate medical and nursing programs in Colombia. The recommendations of the Strengthening the Reporting of Observational Studies in Epidemiology (STROBE) [13] checklist were followed in reporting the study results, which complied with the ethical principles for research and was approved by the Ethics Committee of the Universidad EL Bosque (UEB-2022-519). Informed consent was obtained via email where the research purpose of the survey, confidentiality and data protection were explained.

### Participants

A query within the National Higher Education Information System (SNIES) [14] revealed the registration of 77 nursing and 63 medical programs in Colombia. Undergraduate medical and nursing programs that were active and officially registered with the Colombian Ministry of Education were included. Fifty-eight people were contacted, including program directors and professors in charge of the PC area at various educational institutions, 33 from nursing and 25 from medical programs. Undergraduate medical and nursing programs whose curricula integrated PC competencies were included in the study. The total sample consisted of 48 undergraduate programs: 31 nursing programs and 17 medical programs.

### Data collection tools

The survey used was developed by the research group for the objectives proposed by the study. Two surveys were designed based on the available literature, using as a point of reference the list of PC competencies proposed by the REDCOLEDUPAL [9] (see Supplementary Material 1–3). The initial survey focused on nursing programs, while the second targeted medical programs, aiming to mitigate inclusion and reporting biases. Each survey consists of 18 questions grouped into three domains: (1) Characterization of the context of the institutions, (2) characterization of the structure of PC instruction, and (3) characterization of the CP competencies included in the undergraduate micro curriculum. The surveys were created in Google Forms and sent to each participant’s email address. Data collection was conducted from February 1 to May 30, 2023.

### Data analysis

The data were analyzed using descriptive statistics. Measures of central tendency and dispersion were calculated for quantitative variables, and absolute and relative frequencies were calculated for qualitative variables. Hypothesis tests were then performed using different test statistics depending on the type of variable. The Fisher’s Exact Test test for differences in proportions was used for categorical variables. For quantitative variables, the nonparametric Mann-Whitney U test was used to determine the difference in medians. All tests were performed with an alpha of 0.05 Data were analyzed using R statistical software (version 4.3.1).

## Results

### Context of undergraduate medical and nursing programs in Colombia

The data analysis included 48 undergraduate programs with PC instruction in their curricula: 31 nursing and 17 medical programs. Programs are offered at institutions in 19 of Colombia’s 32 departments, most of which are located in the most densely populated and economically developed cities. Of the total, 77.08% are institutions accredited by the Colombian Ministry of Education with high-quality programs, and 66.67% of the programs are offered by private higher education institutions (See Table 1).

**Table 1:** General characterization of PC education in Colombia

Characteristics	*n* = 48	%
Programs that participated in the survey		
Nursing	31	64.58
Medicine	17	35.42
Type of institution of higher education		
Private	32	66.67
Public	16	33.33
High-quality accreditation by the Colombian Ministry of Education		
Yes	37	77.08
Region where the program is offered		
Andean Region (Antioquia, Bogotá D.C, Quindío, Boyacá, Risaralda, Tolima, Santander, Cundinamarca, Caldas, Huila)	30	62.5
Pacific Region (Valle de Cauca, Nariño, Cauca)	9	18.75
Caribbean Region (Bolívar, Magdalena, Atlántico, Cesar)	6	12.50
Orinoco region (Meta)	3	6.25

### Characterization of PC instruction in undergraduate medical and nursing programs

Undergraduate nursing programs had a median of 163 credits, and medical programs had a median of 284 credits (one academic credit equals 48 h of student work). PC competencies are taught face-to-face in 89.13% of programs, specifically in 93.55% of nursing and 82.35% of medical programs (See Table 2).

PC instruction is distributed in different ways in the curricula. In 41.67% of nursing and medical programs, PC competencies are integrated transversally across the curriculum, which means they are taught in various subjects throughout the course of study. Likewise, 31.25% of the programs have elective courses on PC, and 27.08% have mandatory courses specifically on PC within their curricula. Regarding clinical rotations, 75% of nursing and medical programs did not include a specific PC clinical rotation and those that did it require a median of 60 h (standard deviation ± 58 h). In terms of hours of PC instruction, the average is 81 h for nursing programs and 57.6 h for medical programs. In 72.92% of the programs, no continuing education programs in PC were found (See Table 2).

**Table 2:** Characteristics of PC instruction in undergraduate medical and nursing programs in Colombia

Category		Total		Nursing		Medicine		*P* value
		*n*	%	*n*	%	*n*	%	
Type of courses	Specific curriculum courses *	20	41.67	12	38.71	8	47.06	**0.69**
	Elective course	15	31.25	11	35.48	4	23.53	
	Mandatory course	13	27.08	8	25.81	5	29.41	
Mode of instruction	On-campus face-to-face teaching	43	89.58	29	93.55	14	82.35	**0.20**
	Technology-mediated face-to-face teaching	2	4.17	1	3.23	1	5.88	
	Virtual distance teaching	1	2.08	1	3.23	0	0	
	Other	2	4.17	0	0	2	11.76	
PC clinical rotations	No	36	75	25	80.65	11	64.71	**0.22**
	Yes	12	25	6	19.35	6	35.29	
Continuing education	No	35	72.92	22	70.97	13	76.47	**0.68**
	Yes	13	27.08	9	29.03	4	23.53	

* PC contents are integrated transversally across the curriculum

The nonparametric Mann-Whitney U test was used to determine if there was a difference between the number of credits and the incorporation of PC instruction in undergraduate medical and nursing programs (*p* = 0.007), and a statistically significant difference was found being higher in medical students median 284 Interquartile Range (RIQ) ( 260–300) and nursing students median 163 RIQ ( 152–170). No statistical difference was found between the type of course and the number of credits of the program (*p* = 0.23), nor between the number of theoretical hours dedicated to PC training for nursing (*p* = 0.86) and the number of theoretical hours dedicated to PC training for medicine (*p* = 0.47). PC is taught in nursing programs between semesters IV and VIII (second and fourth year), while in medical programs, PC is taught between semesters V and XII (third and sixth year), with a higher frequency in semester IX (fifth year) (41.2%).

### Characterization of PC competencies taught in nursing and medicine programs

For undergraduate nursing students, the PC competencies most commonly taught were defining PC and understanding the history of PC, identifying the most common symptoms associated with advanced or terminal illness, and understanding pharmacologic and non-pharmacologic pain management. Euthanasia and bio-legal aspects of PC are the least addressed competencies in the nursing curricula. For undergraduate medicine programs, the most frequent competencies were understanding pharmacological and non-pharmacological pain management, identifying PC needs, and knowing the definition and psychological aspects of advanced diseases. The least addressed competencies in medical curricula are the use of the subcutaneous route and pediatric PC (See Table 3).

**Table 3:** PC competencies of nursing and medical programs in Colombia

Category	PC competencies in Colombia			
	Nursing		Medicine	
Definition and basics	PC definition PC history Use of PC assessment scales PC conceptual model PC quality indicators	31 (100%) 30 (96.8%) 24 (77.4%) 19 (61.3%) 7 (22.6%)	PC definition PC history	14 (82.4%) 7 (41.2%)
Identification and symptom control	Pharmacological management of pain Symptom identification Non-pharmacological pain management Opioid pharmacology Problem detection and palliation Pediatric PC	30 (96.8%) 28 (90.3) 27 (87.1%) 18 (58.1%) 17 (54.8%) 7 (22.6%)	Pharmacological pain management Identification of PC needs Non-pharmacological management of pain Pharmacological management of other symptoms Regulation for controlled medications Opioid pharmacology Pediatric PC	17 (100%) 16 (94.1%) 15 (88.2%) 10 (58,8%) 9 (52.9%) 9 (52.9%) 4 (23.5%)
End-life care	End-of-life signs Subcutaneous medications	23 (74.4%) 23 (74.4%)	Treatment withdrawal or withholding. Palliative sedation Subcutaneous route of administration	12 (70,6%) 11 (64.7%) 5 (29.3%)
Ethical and legal issues	Ethics and advance directives PC legal framework Spiritual aspects of PC Euthanasia and bio-legal aspects of PC	25 (80.6%) 21 (67.7%) 23 (74.4%) 1 (3.2%)	Ethics and advance directives PC legal framework	12 (70.6%) 11 (64.7%)
Psychosocial and spiritual issues	Psychology of chronic diseases, death, and grief. Family aspects of the patients Caregiver burnout Therapeutic communication Social aspect of PC	26 (83.9%) 25 (80.6%) 17 (54.8%) 19 (61.3%) 16 (51.6%)	Psychology of chronic diseases, death, and grief. Social aspects of advanced disease Therapeutic communication Spiritual aspects of advanced disease Family aspects of the patients Caregiver burnout	13 (76.5%) 6 (35.3%) 10 (58.8%) 10 (58.8%) 9 (52.9%) 8 (47.1%)

The difference of proportions with the Fisher’s Exact Test. test showed that there was no statistical difference between the proportions of the medical and nursing programs in terms of the PC competencies shared by both curricula, which are, legal framework of PC; psychological, spiritual, and social aspects of chronic disease; grief and loss; therapeutic communication; and ethics, dilemmas, and advance directives. A statistically significant difference was found in PC definition and philosophy, history and the approach to subcutaneous route management between nursing and medical programs being higher in proportion for nursing students 74% compared 29% in medical students (see Table 4).

**Table 4:** Comparison of PC competencies in undergraduate nursing and medical programs in Colombia

PC competencies	Nursing %	Medicine %	*p*
PC definition and philosophy	100	82	0.03
PC history	96	41	< 0.001
PC legal framework	67.7	64.7	1
Psychological aspects of chronic disease, grief, and loss	83.9	76.5	0.7
Spiritual aspects of advanced disease	74.4	58.8	0.33
Social aspects of advanced disease	51.6	35.3	0.36
Therapeutic communication (establishing priorities, prognosis, needs of the last days/weeks of life)	61.3	58.1	0.76
Ethics, dilemmas, and advance directives	80.6	70.6	0.48
PC research	29	0	0.17
Subcutaneous route of administration	74	29	0.005

## Discussion

It is more frequently observed that PC competencies are integrated transversally across medical and nursing curricula, which means that students in both programs take PC subjects at different points during their education. The study identified a low availability of PC clinical rotations and a greater emphasis on conceptual competencies, which may limit students’ ability to develop the practical skills needed to provide quality palliative care. Overall, the study results suggest that improvements are needed in PC education in undergraduate medical and nursing programs in Colombia. These improvements are essential to ensure that students and health professionals acquire the competencies to provide quality palliative care.

### Structure of PC instruction

Martins Pereira et al. [15] found in their study that in 14 European countries, PC is not a mandatory course in undergraduate nursing programs. In contrast, regarding medical programs, Carrasco et al. [16] report in their study that a PC course is mandatory in universities in six European countries; however, in 14 other countries, PC courses are not included in curricula of medical programs. Similarly, in some medical programs, PC courses usually start as electives and later become mandatory courses [17]; according to Eychmüller et al. [18], all medical schools have increased PC contents, teaching staff, and hours of instruction in recent years.

The Latin American Association of Palliative Care (ALCP) recommends including a mandatory PC course in undergraduate programs in medicine, psychology, nursing, and social work and developing validated models to provide structured, homogeneous, and organized training [19]. In 2012, Cuba and Uruguay included a mandatory PC subject in the undergraduate curricula of all medical schools, and 12 other countries, including Colombia, offered it as an elective; in terms of nursing programs, there were 12 Latin American countries with PC lectures, but the way the competencies were developed was not specified [20]. Regarding the implementation of the ALCP recommendations by 2022, Chile reported that 91% of nursing and medical schools already included mandatory training in PC in clinical education before internships and PC content in courses with theory-centered teaching methods [21].

Colombia has also made progress in implementing ALCP recommendations. In 2012, less than 5% of medical schools had incorporated PC content into their undergraduate curricula [20]; by 2021, a descriptive analysis reported that ten medical and 23 nursing programs had specific undergraduate PC instruction [22]. In July 2022, Law 2241 was enacted to promote the integration of the PC essentials in the curriculum of undergraduate programs in health sciences, psychology, and social work [23]; however, it did not make PC instruction mandatory.

In our study, PC instruction is predominantly distributed across the programs’ curricula, followed by PC instruction in elective courses and, less frequently, mandatory courses. Undergraduate courses favor face-to-face teaching modalities and theoretical competencies over practical ones; technology-facilitated learning still needs to be improved in our context. On average, the integration of PC instruction into the programs takes place between the second and fourth year (IV and VIII semesters) for nursing and between the third and sixth year (V and XII semesters) for medicine. Other authors also report the inclusion of PC subjects between the third and fourth year of undergraduate education [24]. PC instruction during the academic program averages 81 h for nursing and 57.6 for medicine. These data exceed the minimum of 40 h of PC instruction recommended by the EAPC [10]. They are also higher than the 15 to 28 h of PC instruction reported by other authors for the total duration of the program [10, 18, 24]. In addition, we found that most PC instruction is classroom-based and focused on theory, and only 25% of programs include PC clinical rotations. It is important to recognize that, to improve the learning and performance of healthcare professionals in PC, it is imperative to combine theoretical learning with practical experiences [25].

### Teaching PC competencies in undergraduate programs

A list of PC competencies to be taught was proposed for Colombia in 2015 [8]. Since 2016, six categories of PC competencies have been determined in undergraduate medical and nursing programs. These categories include the definition and principles of PC, identification and control of symptoms, end-of-life care, ethical and legal issues, psychosocial and spiritual issues, and teamwork [7]. The IAHPC [9] and EAPC [10] both recommended these competencies. In general, the curriculum should have a percentage distribution of PC competencies as follows: Basics of PC, 5%; pain and symptom management, 50%; psychosocial and spiritual aspects, 20%; ethical and legal issues, 5%; communication, 15%; and teamwork and self-reflection, 5% [10].

In our study, competencies in clinical aspects of symptom identification and management and psychosocial and spiritual issues are the most frequently addressed in curricula. Physical and psychological symptomatology are the most common competencies compared to communication aspects or the basics of PC. For nursing, the most addressed competencies are the definition of PC, the legal framework of PC, and the identification of common symptoms associated with advanced or terminal disease. For medicine, pharmacologic and non-pharmacologic pain management and identification of PC needs predominate. Symptom management competencies show that pain is the primary symptom addressed in medical curricula, ignoring the importance of educating students about other common physical symptoms that can be managed with PC interventions. On the other hand, nurses need more education in subcutaneous route management as a procedural skill since the subcutaneous route is widely used in PC patients. In both programs, learning about the psychoaffective symptoms of chronic diseases and bereavement was relevant even more than learning about symptom management and clinical or pharmacological issues. Communication skills are taught in 60% of the programs, which is a low percentage considering that it is a key skill recommended by the different guidelines [8–11].

The available literature confirms that symptom management is a predominant issue addressed and that pain is the most commonly treated symptom [26]. Aspects related to communication (26–27), basics of PC, end-of-life care, bereavement, ethics, cultural beliefs, the health care system, institutional issues, and community, psychosocial, and spiritual care are also addressed in the curricula [28]. Both nursing and medicine undergraduate curricula integrate PC competencies that are highly aligned with current recommendations [7–10].

There is no evidence in our data that indicate that higher education institutions are developing curricula to meet the specific needs of the population, beyond the recommended PC competencies for undergraduate programs. Educational strategies and modalities for medical education do not vary significantly and tend to focus on practical experience in clinical rotation settings.

Recently, academic programs in the health sciences have adopted processes to demonstrate the desired learning outcomes of competency-based education, and this has led to the emergence of Entrustable Professional Activities (EPAs), which are key tasks that health professionals must know how to perform and that serve as observable and meaningful assessment points [29, 30]. We did not identify this approach in the teaching models reported by the programs that participated in the survey.

### Study limitations

This study includes 27% of the country’s medical and 40% of the nursing programs. The data reported are calculated based on the programs that participated in the survey, which means that the results cannot be systematically generalized. The time limit for submitting completed surveys may have limited the sample size. The limited availability of previous local information on the subject under study prevents comparing the data obtained with country-specific information.

## Conclusions

PC education in Colombia’s undergraduate medical and nursing programs favors theoretical competencies over practical ones. In addition, most programs integrate PC competencies across subjects throughout the curriculum rather than bringing them together in a specific mandatory course. This situation highlights the need for curricular improvements to ensure that healthcare students and professionals develop the necessary competencies to provide quality palliative care. It is necessary to establish academic processes to demonstrate the desired learning outcomes of competency-based education and to evaluate the impact of incorporating PC instruction into undergraduate programs.

### Electronic supplementary material

Below is the link to the electronic supplementary material.

Supplementary Material 1

Supplementary Material 2

Supplementary Material 3

## Data Availability

All data generated or analyzed during this study are included as supplementary information files or are available from the corresponding author on reasonable request: sanchezcmiguel@unbosque.edu.co.

## Abbreviations

REDCOLEDUPAL: Colombian Palliative Care Education Network
OCCP: Colombian Palliative Care Observatory
EAPC: European Association for Palliative Care
IAHPC: International Association for Hospice & Palliative Care
SNIES: National Higher Education Information System
ALCP: Latin American Association of Palliative Care
PC: Palliative Care
STROBE: Strengthening the Reporting of Observational Studies in Epidemiology
WHO: World Health Organization
