# Supplementary material for: Palliative care education in undergraduate medical and nursing programs in Colombia: a cross-sectional analysis
Source: BMC Palliat Care. 2024 Jun 13;23:149. doi: 10.1186/s12904-024-01477-5 (PMC11170879; doi:10.1186/s12904-024-01477-5)
Supplement: Supplementary file 1 — Supplementary Material 1 [file 12904_2024_1477_MOESM1_ESM.docx]

**Appendix 2.** REDCOLEDUPAL’s palliative care competencies for nursing programs.

| Define palliative care, its philosophy, and basic principles. |
| --- |
| Understand the hospice care philosophy and the principles and history of palliative care. |
| Identify the most common symptoms associated with advanced and/or terminal illness. |
| Detect care needs for symptom control in advanced and/or terminal illness. |
| Recognize pharmacologic and non-pharmacologic principles for symptom management and control. |
| Develop skills in the safe administration of subcutaneous medications. |
| Develop skills in the use of palliative care assessment scales. |
| Know the appropriate measures to detect, control, or palliate the most common clinical problems and emergencies that occur in the advanced stages of a disease. |
| Identify the signs of the last days of life and provide specific care associated with this phase of a disease. |
| Recognize the adaptive responses of the patients and their families to terminal illness. |
| Identify coping strategies when facing the presence of death. |
| Identify patients’ and families’ resources for coping with an advanced and/or terminal illness. |
| Develop cross-cultural caregiving skills by identifying religious and cultural implications and patients’ and families’ values and beliefs when facing a terminal illness and death. |
| Recognize the most common nursing interventions for families with a relative in palliative care to help prevent ‘*claudicación familiar’* (family surrender). |
| Identify the specific aspects for establishing communication processes in the different situations of patients and their families facing a terminal illness. |
| Develop skills in delivering bad news to ensure therapeutic relationships. |
| Identify the nurse’s responsibilities in the care of palliative care patients and those of the primary caregiver and family. |
| Recognize public policies related to palliative care in the national territory. |
| Design, plan, implement, and evaluate nursing care plans and programs based on transdisciplinary decision-making in the care of terminally ill patients and their families. |
| Recognize the importance of working in collaboration with other professionals to improve the quality of nursing care. |
