# Supplementary material for: Palliative care education in undergraduate medical and nursing programs in Colombia: a cross-sectional analysis
Source: BMC Palliat Care. 2024 Jun 13;23:149. doi: 10.1186/s12904-024-01477-5 (PMC11170879; doi:10.1186/s12904-024-01477-5)
Supplement: Supplementary file 2 — Supplementary Material 2 [file 12904_2024_1477_MOESM2_ESM.docx]

**Appendix 1.** REDCOLEDUPAL’s palliative care competencies for medical programs.

| Define palliative care |
| --- |
| Understand the health care system and the place and organization of palliative care. |
| Identify who needs palliative care. |
| Identify when it is appropriate to provide palliative care. |
| Know the basics of symptom management. |
| Know the palliative care clinical record. |
| Know the scales for assessing symptoms, quality of life, and functional independence. |
| Know the regulations for prescribing controlled medications and international agreements related to controlled drugs. |
| Recognize nociceptive pain mechanisms (bone, soft tissue, visceral pain) and neuropathic pain mechanisms. |
| Recognize the characteristics of chronic pain. |
| Understand the concept of “total pain.” |
| Know the principles of pharmacological treatment (WHO analgesic ladder). |
| Know the pharmacokinetics and pharmacodynamics of opioids, non-opioids, and adjuvant analgesics. |
| Understand opioid titration and rotation used for pain management. |
| Identify routes of drug administration and their indications, as well as alternative routes when oral administration is not possible. |
| Recognize other pharmacologic and non-pharmacologic options in pain management. |
| Understand the pathophysiology and know the medications indicated for the treatment of nausea, vomiting, and constipation. |
| Understand the pathophysiology and know the medications indicated for treating dyspnea and cough. |
| Understand the pathophysiology and know the medications indicated for treating insomnia, delirium, and anxiety. |
| Understand the pathophysiology and know the medications indicated for treating symptoms of mucositis and skin ulcers. |
