# Supplementary material for: Palliative care education in undergraduate medical and nursing programs in Colombia: a cross-sectional analysis
Source: BMC Palliat Care. 2024 Jun 13;23:149. doi: 10.1186/s12904-024-01477-5 (PMC11170879; doi:10.1186/s12904-024-01477-5)
Supplement: Supplementary file 3 — Supplementary Material 3 [file 12904_2024_1477_MOESM3_ESM.docx]

**Characterization of Palliative Care education at undergraduate level in nursing in Colombia OCCP**

Dear participant, we appreciate your willingness and time to answer this simple and brief questionnaire. In the following form you will find a series of questions related to characteristics about the education offered in the Nursing program at your educational institution. Your response is of utmost importance to us, remember that the responses recorded will be anonymous. If you have any questions you can contact us via email [occp@unbosque.edu.co](mailto:occp@unbosque.edu.co). We appreciate your contribution by answering the following questionnaire. Strengthen education in palliative care, a commitment to comprehensive care.

1. **Institution data**

**Type of institution**

- Private
- Public

**Where in the country is the institution locate**

- Amazonas
- Antioquia
- Arauca
- Atlantico
- Bogotá D.C
- Bolivar
- Boyacá
- Caldas
- Caqueta
- Casanare
- Cauca
- Cesar
- Chocó
- Cordoba
- Cundinamarca
- Guainía
- Guaviare
- Huila
- La Guajira
- Magdalena
- Meta
- Nariño
- North of Santander
- Putumayo
- Quindío
- Risaralda
- San Andrés
- Santander
- Sucre
- Tolima
- Valle del Cauca
- Vaupes
- Vichada

**Does the institution have high-quality accreditation?**

- Yes
- No

**Indicate the number of total credits of the Nursing program:**

**Does the institution incorporate palliative care in its curriculum?**

Incorporation in any modality such as: mandatory subject, elective or transversal content within different subjects.

- Yes
- No

**Modality through which palliative care education is incorporated**

Indicate the modality through which palliative care content is provided.

- Mandatory subject (Subject that must be taken by all students)
- Elective subject (Subject that is chosen by students voluntarily)
- Transversal contents (contents that are taught in the different subjects)

**Stage in which the subject in palliative care is incorporated**

Indicate the semester in which palliative care training is incorporated.

- I
- II
- III
- IV
- V
- VI
- VII
- VIII
- IX
- X

1. **Palliative care training**

**Indicate the modality in which palliative care content is offered.**

- Remote teaching
- In person on campus
- In-person mediated by technology.

**Indicate the number of theoretical hours dedicated to training in palliative care**

If your answer was transversal content as a modality of incorporation, the hours dedicated to content after palliative care must be added.

**Indicate the topics addressed in the training process**

- Definition of palliative care
- Legal framework of palliative care.
- History of palliative care
- Conceptual models of nursing care in palliative care
- Identification of the most common symptoms associated with advanced and/or terminal illness
- Pharmacological pain management
- Non-pharmacological pain management
- Subcutaneous drug administration
- Use of rating scales in palliative care
- Timely measures to detect, control and alleviate clinical problems and the most common emergency situations that occur.
- Signs and symptoms of end of life
- Coping strategies in the presence of death.
- Psychological aspects of chronic illness, grief and loss
- Spiritual aspects of palliative care
- Social aspects of palliative care
- Family aspects such as adaptation, emotional stages of the patient and his family
- Identification and prevention of "caregiver burnout"
- Therapeutic communication (establishing priorities, prognosis, needs of the last days/weeks of life)
- Pediatric palliative care
- Pharmacokinetics and pharmacodynamics of opioid, non-opioid, and adjuvant analgesic medications
- Collaborative work with other professionals to improve the quality of nursing care
- Basic quality indicators in a palliative care program
- Ethics, dilemmas, advance directives.
- Palliative care research

**Does the institution offer clinical practice rotation in palliative care?**

- Yes
- No

1. **Continuing training in palliative care**

**Does the institution offer continuing training for students in the program?**

- Yes
- No

1. **How is continuing training in palliative care offered?**

**How does the institution offer continuing training in palliative care for students in its program?**

- Seminar
- Congress
- Workshop
- None

1. **Homologation process for the palliative care subject**

**Can the student take the subject at another institution and carry out the homologation process for it?**

- Yes
- No

1. **Teacher training**

**Do teachers have specific education or training in palliative care?**

- Yes
- No

**How many teachers are specifically trained in palliative care? (**Specific postgraduate training that includes the name Palliative Care)

**Indicate the postgraduate training name of each of them**

1. Teaching Specialization

2. Teaching mastery

3. Teaching Doctorate

4. Teaching Postdoc

5. More than two levels of training

At this point you have finished the questionnaire.

Your response is very important to us. We appreciate your participation. You can proceed to send your responses.
